# Hospital admission and mortality rates for non-Covid diseases among residents of the long-term care facilities before and during the pandemic: a cohort study in two Italian regions

**DOI:** 10.1007/s10389-023-01925-1

**Published:** 2023-05-16

**Authors:** Sara Mazzilli, Giuditta Scardina, Francesca Collini, Silvia Forni, Giulio Gianolio, Lucia Bisceglia, Pier Luigi Lopalco, Antonio Chieti, Graziano Onder, Nicola Vanacore, Guglielmo Bonaccorsi, Fabrizio Gemmi, Lara Tavoschi

**Affiliations:** 1 Scuola Normale Superiore, Pisa, Italy; 2 Department of Translational Research and New Technologies in Medicine and Surgery, University of Pisa, Pisa, Italy; 3 Quality and Equity Unit, Regional Health Agency of Tuscany, Florence, Italy; 4 Strategic Regional Health and Social Agency of Puglia (AReSS Puglia), Bari, Italy; 5 Department of Biological and Environmental Sciences and Technology, University of Salento, Lecce, Italy; 6 Department of Cardiovascular, Endocrine-Metabolic Diseases and Aging, National Institute of Health, Rome, Italy; 7 National Centre for Disease Prevention and Health Promotion, National Institute of Health, Rome, Italy; 8 Department of Health Science, University of Florence, Florence, Italy

**Keywords:** Long-term care facilities, Covid-19 pandemic, Hospital admission, Mortality risk

## Abstract

**Aim:**

Long-term-care facility residents are a vulnerable population who experienced reduced healthcare access during the pandemic. This study aimed to assess the indirect impact of the COVID-19 pandemic, in terms of hospitalisation and mortality rates, among this population in two Italian Regions, Tuscany and Apulia, during 2020 in comparison with the pre-pandemic period.

**Subject and methods:**

We conducted a retrospective cohort study on people residing in long-term-care facilities from 1 January 2018 to 31 December 2020 (baseline period: 1 January 2018–8 March 2020; pandemic period: and 9 March–31 December 2020). Hospitalisation rates were stratified by sex and major disease groups. Standardised weekly rates were estimated with a Poisson regression model. Only for Tuscany, mortality risk at 30 days after hospitalisation was calculated with the Kaplan–Meier estimator. Mortality risk ratios were calculated using Cox proportional regression models.

**Results:**

Nineteen thousand two hundred and fifty individuals spent at least 7 days in a long-term-care facility during the study period. The overall mean non-Covid hospital admission rate per 100 000 residents/week was 144.1 and 116.2 during the baseline and pandemic periods, with a decrease to 99.7 and 77.3 during the first (March–May) and second lockdown (November–December). Hospitalisation rates decreased for all major disease groups. Thirty-day mortality risk ratios for non-Covid conditions increased during the pandemic period (1.2, 1.1 to 1.4) compared with baseline.

**Conclusion:**

The pandemic resulted in worse non-COVID-related health outcomes for long-term-care facilities’ residents. There is a need to prioritise these facilities in national pandemic preparedness plans and to ensure their full integration in national surveillance systems.

**Supplementary information:**

The online version contains supplementary material available at 10.1007/s10389-023-01925-1.

## Introduction

People living within long-term care facilities (LTCFs) typically suffer from multiple long-term conditions, cognitive impairment, and medical and social vulnerabilities (European Centre for Disease Prevention and Control 2021). Due to characteristics of this population and structural or organisational features of the facilities — such as staff working in multiple facilities and high rates of staff turnover (Mukamel et al. 2009) — risks of contracting Covid-19 or developing subsequent severe outcomes, especially before the introduction of the Covid-19 vaccination, were high among LTCF resident population (Giddings et al. 2021; McMichael et al. 2020). In a report published in May 2020 by the European Centre for Disease Prevention and Control (ECDC), it was estimated that deaths among LTCF residents accounted for 37–66% of all Covid-19 related deaths in EU/EEA countries, depending on the country. No data was provided on the Italian LTCF situation (ECDC Public Health Emergency Team 2020).

In Italy, the first confirmed autochthonous cases of Covid-19 were detected in Lombardy, a region in the North of Italy, in late February 2020, with subsequent spread to other regions at different times and rates (Bezzini et al. 2022; Gatto et al. 2020). During the first phase of the pandemic, the real number of Covid-19 cases among LTCF residents was difficult to assess due to the lack of a surveillance system specifically for LTCFs and context and time-specific features (e.g., low or absent testing capacity and undertrained staff (ECDC 2021). To fulfil these gaps, in March 2020, the Italian National Health Institute dedicated a survey to the issue. A dramatic picture emerged from it, with LTCFs reporting high infection rates (Lombardo et al. 2021). In addition, results from studies conducted at the national and local levels in 2020 suggest that the impact of the pandemic in terms of mortality among LTCF residents was large (de Girolamo et al. 2020; Tramarin et al. 2021). In particular, a study evaluating mortality rates of older adults in LTCF across different Italian regions during the first 4 months of 2020 highlighted that risk of death among LTCF residents increased about 4 times during the study period when compared to the same period in the previous years. Moreover, mortality rates of LTCF residents were significantly higher when compared to age-specific mortality rates of the general population (de Girolamo et al. 2020). Another national study highlighted that the Covid-19 incidence rate does not fully explain the differences of mortality impact among different regions (Amore et al. 2021).

Although less broadly investigated, it is expected that people living within LTCFs were also more likely to develop worse health outcomes for non-Covid conditions during the first phase of the pandemic. The non-medical measures enforced by Governments during the pandemic, including dramatic restrictions on individual liberties and prioritisation of healthcare services to reduce risks of system collapse (Bodilsen et al. 2021; Maringe et al. 2020), influenced access to healthcare services and hospital admissions for the entire population. In particular, in Italy, in March 2020, the Government asked regions to postpone outpatient and hospital scheduled activities that were not considered of high priority during the emergency, with a progressive re-activation from June 2020 (Direzione Generale della Programmazione Sanitaria 2020).

As highlighted by a systematic review by Moynihan et al. on 20 countries worldwide, including Italy, such restrictive measures, along with fear of becoming infected or inability to access care, resulted in an overall decrease of one-third of healthcare utilisation by the general population (Moynihan et al. 2021). No information was provided on LTCF resident population.

In the literature, the effects of the pandemic on non-communicable diseases (NCDs) have been extensively studied. In the UK, according to a retrospective cross-sectional study, total admissions and emergency department attendances for selected cardiovascular diseases dropped between 31% and 88% during the first lockdown in 2020 in comparison with 2019 (Ball et al. 2020; Mafham et al. 2020) and excess in acute cardiovascular mortality (+8%) was observed in England and Wales during the first semester of 2020 compared with the expected historical average in the same period of the year (Banerjee et al. 2021; Poon et al. 2020; Wu et al. 2021). Similar results have been observed in the USA, with a national increase in deaths by ischemic heart diseases and hypertensive diseases in the first semester of 2020 compared with 2019 (Wadhera et al. 2021). Further studies conducted worldwide, including in Italy (Cannata et al. 2022; Fondazione GIMBE Bologna 2021), investigating the indirect impact of the pandemic on a vast range of NCDs — especially cardiovascular diseases and cancer — showed similar results, with a decrease in hospital admissions for NCDs and an increase in disease burden after the onset of the pandemic compared to the pre-pandemic period (Gadsden et al. 2022; Mak et al. 2022; Maringe et al. 2020; Pécout et al. 2021; Richards et al. 2020).

Although many studies have examined the impact of the pandemic on NCDs among the general population, investigations on LTCF resident population are scarce. A study conducted among Canadian LTCF residents during the first 6 months of the pandemic, observed that hospital admissions for non-Covid-19 diseases dropped by 27% during the study period in comparison with the same period in 2019, with the largest decrease in hospital transfers for chronic conditions requiring a doctor’s order to seek hospital care, such as heart failure and chronic respiratory conditions, while deaths for all causes increased by 19% in comparison with the average 5-year period (Betini et al. 2021).

Studies on the effects of the pandemic on the utilisation of healthcare services for non-Covid-19 diseases among LTCF residents in Italy are missing, and those on mortality for all causes limited. Availability of health data with regard to residents in LCTFs is limited and largely related to regional areas, as a consequence of decentralisation of healthcare services in the country and the coexistence of private and public LTCFs.

This study is a retrospective cohort study aimed to assess the indirect impact of the COVID-19 pandemic on the health outcomes of LTCFs residents in two Italian regions, Tuscany and Apulia (Central and Southern Italy). Hospital admission and mortality rates among LTCF residents were monitored during the 1st year of the pandemic and in the pre-pandemic period to identify significant changes.

## Methods

### Demography and healthcare services in the two regions

On 31 December 2020, there were 3,692,865 residents in Tuscany and 3,933,777 residents in Apulia. The distribution by sex was similar (females > 51% in both regions), while population in Tuscany was slighter older (mean age of 47 years in Tuscany and 45.4 in Apulia), with people over 65 representing about 25.7% of the whole population in Tuscany and 23.1% in Apulia. In 2019, people with at least one chronic disease were 40.9% and 40.4% of the population in Tuscany and Apulia, with the most frequent chronic conditions being hypertension (17.2% and 18.8%), arthrosis and arthritis (15.5% and 17.0%), allergic diseases (11.4% and 11.8%) and osteoporosis (7.4% and 9.4%) in both regions, followed by chronic bronchitis for Tuscany (5.4%) and diabetes for Apulia (7.1%) (Osservatorio Nazionale sulla Salute nelle Regioni Italiane 2019).

In the decade before the pandemic, hospital care went through a nationwide reshaping with the aim of increasing appropriateness and safety of care. De-hospitalisation and implementation of community healthcare was at the core of this reorganisation, with reduction in hospitalisations rate and number of hospital beds (Agenzia Regionale di Sanità Toscana 2021). In 2020, the hospital network in Tuscany included 39 public and 21 accredited private care institutions, for a total of 10,254 beds in ordinary inpatient care (2.78 beds per 1.000 inhabitants, lower than the Italian average of 3.1). In Apulia, the network included 33 public and 26 private institutions, for a total of 11,565 beds in ordinary inpatient care and 2.93 beds per 1,000.

In 2019, the standardised rates of hospitalisation for all causes by age and gender per 1,000 inhabitants in Tuscany and Apulia were among the lowest in Italy (120.4 and 114.6 respectively), confirming the downward trend already observed since 2013. The number of beds for non-self-sufficient elderly people in LTCFs in Tuscany was significantly higher than in Apulia in 2016 (146.0 beds compared to 74.2 per 10,000 inhabitants), with the number of residents in LCTFs per 1,000 inhabitants being respectively 13.5 and 7.1. Also, the rate of people over 65 who benefited from home care and community care services per 1,000 inhabitants in 2019 was 32.8 in Tuscany and 19.4 in Apulia (the overall Italian average was 28.1) (Osservatorio Nazionale sulla Salute nelle Regioni Italiane 2019).

### Setting and study population

The Italian healthcare system is a regionally based national health service (NHS). The system is organized into three levels: national, regional, and local. The national level is responsible for establishing the general objectives and fundamental principles of the NHS. The regions and autonomous provinces (R&AP) (19 regions and two autonomous provinces) are then responsible for organising and delivering health care (Ferré et al. 2014). In this scenario, the R&AP are in charge of organising the long term-care facilities at the local level based on national guidelines.

Long-term care facilities have been established in Italy in the mid-1990s, after the release in 1989 of a governmental policy act for the creation of residential health care facilities for the elderly (Il Presidente del Consiglio dei Ministri 1989). With the release of two more decrees in 2001 (Il Presidente del Consiglio dei Ministri 2001a), the Government endorsed the role of social protection of the LTCFs, promoting integration between social and health care services within the facilities, and included assistance in LTCFs among those essential services that the NHS guarantees to non-self-sufficient people who do not have the possibility to care for themselves at home (Il Presidente del Consiglio dei Ministri 2001b). LTCFs can be administered by the public (NHS or the municipality) or by the private sector (alone or in affiliation with the NHS). The percentage of LTCFS administered by public or private sector can vary widely among regions.

In 2020, in Tuscany, there were 312 LTCFs with 13,997 beds. Of these facilities, 115 were public and 197 private (Regione Toscana 2013); in Apulia, there were 140 LTCFs with 6,937 beds, of which 97% were administered by the private sector (Italian Ministry of Health 2013). Most private facilities are contracted by the public system that partially subsidise the cost of resident stay. In Italy, the state provides all residents with free and unrestricted access to healthcare (La Camera dei Deputati e Il Senato della Repubblica 1978). Data are recorded prospectively in regional registries, allowing longitudinal surveillance of the entire population. This cohort study included individuals residing in the LTCFs from 1 January 2018 to 31 December 2020. The individuals included in the study were residing for at least 7 consecutive days in the LTCFs of Tuscany and Apulia regions during the study period.

### Pandemic stages and preventive measures

In Italy, after the detection of the first locally acquired Covid-19 case in Lombardy on February 20, 2020, the number of cases increased greatly in the following weeks, although unevenly among regions, forcing the government to adopt unprecedented restrictive measures (Il Presidente del Consiglio dei Ministri 2020e, c). In particular, during the first year of the pandemic, the following phases can be defined: (1) first comprehensive national lockdown from 9 March to 3 May (closure of schools and most workplaces, and the implementation of quarantines, border closings, and restriction on public gatherings) (Il Presidente del Consiglio dei Ministri 2020c), (2) gradual reopening phase from 4 May to 14 June (restrictions were gradually rolled back) (Il Presidente del Consiglio dei Ministri 2020d), (3) Few restrictions from 15 June to 7 October (Covid-19 incidence remained low) (Il Presidente del Consiglio dei Ministri 2020a), (4) new restrictions from 8 October to 5 November (Il Presidente della Repubblica 2020), and (5) lockdowns on a regional basis from 6 November until the end of 2020 (closure of regional borders) (Il Presidente del Consiglio dei Ministri 2020b).

The Italian government issued specific provisions aimed at minimising the risk of introducing and transmitting diseases within LTCFs. In particular, with a decree released in April 2020, along with measures regulating the entry of new residents and visits from family members, it was decided to avoid the access of LTCF residents to hospitals as far as possible, even if for specialist visits or diagnostic or therapeutic procedures (Istituto Superiore di Sanità 2020b). In August 2020, the use of telemedicine in delivering care to LTCF residents was promoted, and general practitioners’ access for in-person visits recommended for essential cases only (Istituto Superiore di Sanità 2020a). These national recommendations were endorsed by Tuscany and Apulia, which adapted them to the local context with the implementation of more stringent restrictions if needed.

Due to the fact that Northern Italy was the first and more heavily affected area during the first phase of the pandemic, while Central and Southern Italy were affected later during 2020, our first intention was to select two regions for each area of the country. We asked six Italian regions to participate in the study but only Tuscany and Apulia accepted, due to reported difficulties in using data from the regional registries of people admitted in the public and the contracted private LTCFs.

### Data sources and study period

We conducted a retrospective cohort study by analysing and merging two electronic health records from the Tuscany and Apulia health-care information systems: the regional registries of people admitted in the public and the contracted private LTCFs and the regional hospital discharge dataset (≥ 95% of all LTCF beds available). The study period ran from 1 January 2018 to 31 December 2020. We defined the period between 1 January 2018 and 8 March 2020 as the pre-Covid-19 baseline period, and the period between 9 March and 31 December 2020 as the Covid-19 pandemic period.

The regional registries of people admitted in the LTCFs track age, sex, and LTCF admission and discharge dates. For Tuscany only, this data source also provided data on the vital status of LTCF residents. The regional hospital discharge form dataset includes hospital admission and discharge date, sex, and principal diagnosis of discharge coded according to the World Health Organisation’s international classification of diseases version 9 (ICD-9-CM) (National Center for Health Statistics 2015).

Through a numeric identification code, we linked the registries of LTCF residents with the hospital discharge dataset. All the health data used in the study were anonymous administrative health data routinely collected through the regional health information systems.

The study protocol was approved on October 10, 2021, by the Joint Ethical Committee for research of the Scuola Normale Superiore (Pisa, Italy) and the Sant'anna School for Advanced Studies (Pisa, Italy). Number of approval: 39/2021.

### Outcomes

From 1 January 2018 to 31 December 2020, all hospital admissions of people residing in LCTFs were identified by principal diagnosis codes in the hospital discharge form. These codes were subsequently grouped according to the multi-level diagnosis clinical classification system (CCS) (Agency for Healthcare Research and Quality 2015) which is based on the ICD-9-CM classification. In 2020, in the ICD-9-CM diagnosis group the category “Covid-19” was added.

Only for people residing in LTCFs in Tuscany, we computed the overall weekly average mortality rate per 1,000 LTCF residents before and after the pandemic, and the 30-day mortality risks for hospital admissions investigated overall and according to major disease groups by pandemic period.

### Statistical analysis

We examined overall hospital admission rates stratified by sex and major disease groups. We used a Poisson regression model to estimate standardised weekly hospital admission rates.

We computed hospital admission rate ratios and 95% confidence intervals by using the pre-Covid-19 baseline period (1 January 2018–8 March 2020) as reference. We then repeated the analyses for the two regional study population cohorts.

For the Tuscany region, we assessed mortality risk at 30 days after hospital admission by using the Kaplan–Meier estimator, with censoring at LTCF discharge or end of follow-up whichever occurred first. Mortality risk ratios and 95% confidence intervals were calculated using Cox proportional regression models, and the pre-Covid-19 baseline period (1 January 2018–8 March 2020) as competing risk.

Stata MP version 15 was used for all statistical analysis, and a *p*-value of 0.05 was applied for testing statistical significance.

## Results

A total of 19,250 individuals who spent at least 7 days in a LTCF from 1 January 2018 to 31 December 2020 were included in the study cohort. Of these individuals, 11,806 (median age 87, 28% male) were LTCF residents in Tuscany and 7,444 (median age 84, 29% male) in Apulia. Table 1 shows the weekly average population in the LTCFs in the pre-pandemic period and in the different pandemic phases overall and by region. The number of (non-Covid-19) hospital admission among LTCFs residents in 2018 were 4,883 (3,570 in Tuscany; 1,313 in Apulia), in 2019 were 4,914 (3,667 in Tuscany; 1,247 in Apulia), and in 2020 were 3,456 (2,583 in Tuscany; 873 in Apulia). During the study period, the median age of individuals hospitalised was 85 in Tuscany and 83 in Apulia.

**Table 1:** The weekly average population in LTCFs during the pre-pandemic period and in the different pandemic phases by age and gender, Apulia and Tuscany, 2018–2020

	Baseline before 9 Mar 2020			First national lockdown 9 Mar–3 May 2020			Gradual reopening phase 4 May–14 Jun 2020			Few restrictions 15 Jun–7 Oct 2020			New restrictions 8 Oct–5 Nov 2020			Lockdowns on a regional basis 6 Nov–31 Dec 2020		
Tuscany																		
	Total	F	M	Total	F	M	Total	F	M	Total	F	M	Total	F	M	Total	F	M
65–74	817	378	438	767	355	412	737	344	394	723	331	392	696	318	378	661	301	360
75-84	1,837	1,178	659	1,654	1,075	579	1,568	1,027	542	1,523	990	532	1,460	948	512	1,372	897	475
85–94	2,935	2,364	571	2,712	2,156	555	2,566	2,064	503	2,444	1,979	465	2,322	1,881	441	2,133	1,748	385
< 94	785	713	72	809	740	68	765	703	61	713	654	58	658	599	59	581	532	49
Total	6,374	4,634	1,740	5,941	4,326	1,615	5,636	4,137	1,499	5,402	3,954	1,448	5,135	3,746	1,390	4,747	3,478	1,270
Apulia																		
	Total	F	M	Total	F	M	Total	F	M	Total	F	M	Total	F	M	Total	F	M
	435	201	234	430	199	231	434	202	232	443	202	241	450	206	244	452	206	246
	835	535	300	780	507	273	790	517	273	812	528	284	825	536	289	828	541	287
	1,005	809	196	922	733	190	923	742	181	943	764	179	956	775	181	963	789	174
	121	110	11	122	112	10	119	109	10	117	108	9	119	108	11	116	106	10
Total	2,395	1,656	739	2,255	1,551	704	2,266	1,571	695	2,315	1,602	713	2,350	1,624	726	2,359	1,642	717

### Hospital admission rates

**Fig. 1:**
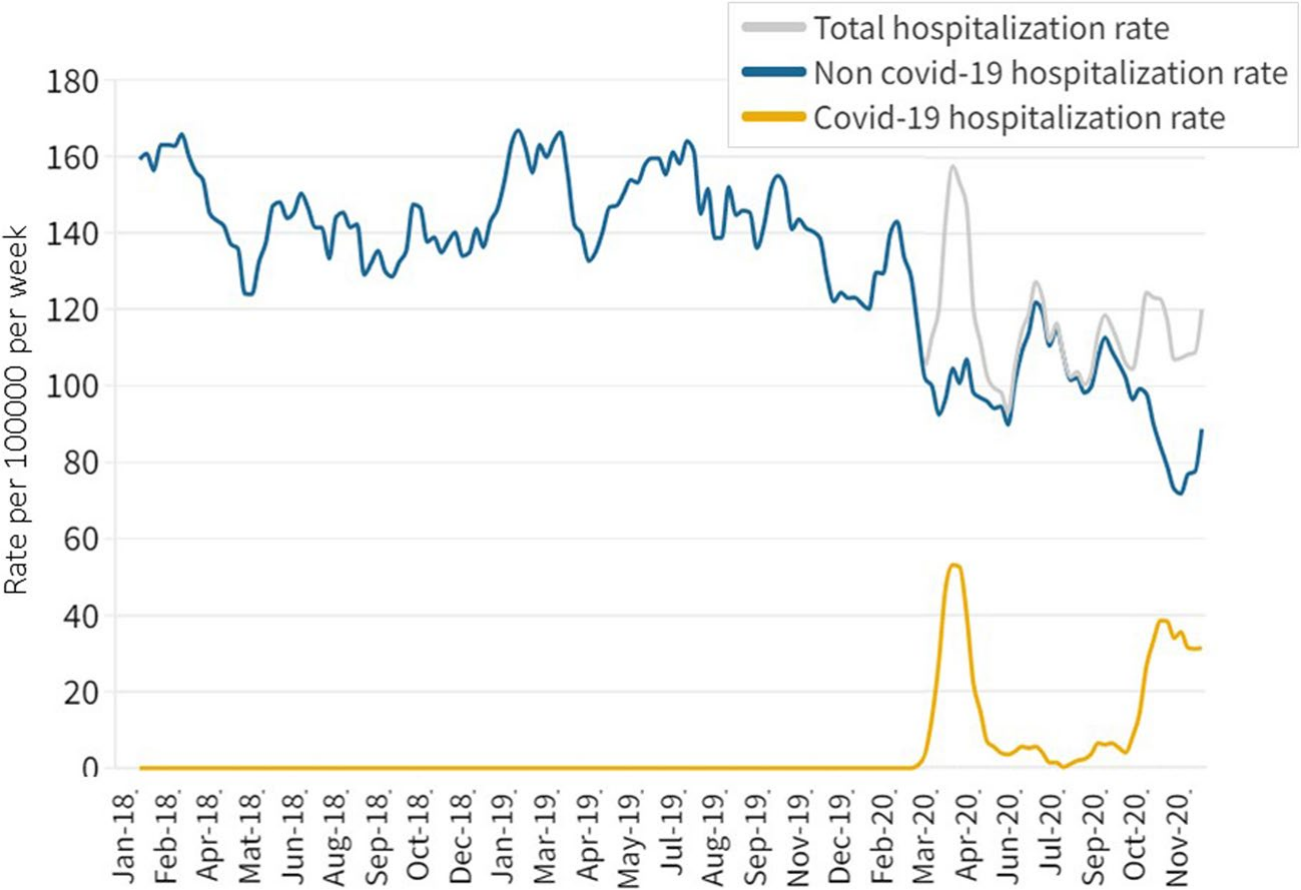
Overall non-Covid-19 (*blue line*) and Covid-19 (*yellow line*) hospital admission rates among LTCFs residents in Apulia and Tuscany, 2018–2020

The overall mean non-Covid-19 hospital admission rate was 144.1 (198.4 males; 123.2 females) per 100,000 LCTF residents/week during the baseline period and 116.2 (138.9 males; 82.5 females) per 100,000/week during the pandemic period (Fig. 1). The overall mean non-Covid-19 hospital admission rate was 155.8 in Tuscany and 114.5 in Apulia during the baseline period, and 123.5 in Tuscany and 56.8 in Apulia during the pandemic period (Supplemental material Table e1 and Table e2). The mean hospital admission rate for non-Covid-19 conditions decreased to 99.7 (rate ratio 0.7; 95% confidence interval (95% CI: 0.6 to 0.8)) during the first national lockdown, 92.1 (0.6; 95% CI: 0.6 to 0.7) during the gradual reopening phase, 108.9 (0.8; 95% CI: 0.7 to 0.8) during the period with few restrictions, 90.1 (0.6; 95% CI: 0.5 to 0.7) during the period with new restrictions, and 77.3 (0.5; 95% CI: 0.5 to 0.6) during regional lockdown phase. The mean hospital admission rate for non-Covid-19 conditions in men decreased to 149.6 (0.8; 95% CI: 0.7 to 0.9) during the first national lockdown, 121.7 (0.6; 95% CI: 0.5 to 0.7) during the gradual reopening phase, 153.3 (0.8; 95% CI: 0.7 to 0.9) during the period with few restrictions, 133.2 (0.7; 95% CI: 0.5 to 0.8) during the period with new restrictions, and 109.5 (0.6; 95% CI: 0.5 to 0.7) during the regional lockdown phase (Supplemental material Fig. 2.A). In female LTCF residents, the mean hospital admission rate for non-Covid-19 conditions decreased to 80.7 (0.7; 95% CI: 0.6 to 0.7) during the first national lockdown, 85.3 (0.7, 0.6 to 0.8) during the gradual reopening phase, 91.6 (0.7; 95% CI: 0.7 to 0.8) during the period with few restrictions, 73.3 (0.6; 95% CI: 0.5 to 0.7) during the period with new restrictions, and 64.2 (0.5; 95% CI: 0.4 to 0.6) during regional lockdown phase (Supplemental material Fig. 2.B) (Table 2). Similar results were obtained when the analysis was repeated by region (Supplemental material Tables 1 and 2).

**Table 2:** Hospital admission rates (per 100,000/week) among LTCF residents in Tuscany and Apulia during the Covid-19 pandemic compared with the pre-pandemic baseline period

	Baseline (reference)	Covid-19 pandemic period				First national lockdown				Gradual reopening phase				Few restrictions				New restrictions				Lockdowns on a regional basis			
	Rate	Rate	RR	(95% CI)		Rate	RR	(95% CI)		Rate	RR	(95% CI)		Rate	RR	(95% CI)		Rate	RR	(95% CI)		Rate	RR	(95% CI)	
Any cause (including COVID-19)	144.1	116.2	0.8	0.8	0.8	133.7	0.9	0.9	1.0	96.5	0.7	0.6	0.7	112.4	0.8	0.7	0.8	124.5	0.9	0.8	1.0	117.3	0.8	0.7	0.9
Any cause (excluding COVID-19)	144.1	98.2	0.7	0.7	0.7	99.7	0.7	0.6	0.8	92.1	0.6	0.6	0.7	108.9	0.8	0.7	0.8	90.1	0.6	0.5	0.7	77.3	0.5	0.5	0.6
Male	198.4	138.9	0.7	0.7	0.8	149.6	0.8	0.7	0.9	121.7	0.6	0.5	0.7	153.3	0.8	0.7	0.9	133.2	0.7	0.5	0.8	109.5	0.6	0.5	0.7
Female	123.2	82.5	0.7	0.6	0.7	80.7	0.7	0.6	0.7	83.5	0.7	0.6	0.8	91.6	0.7	0.7	0.8	73.3	0.6	0.5	0.7	64.2	0.5	0.4	0.6
ICD-9-CM																									
Cardiovascular disease	24.5	15.8	0.6	0.6	0.7	16.4	0.7	0.5	0.8	16.2	0.7	0.5	0.9	17.7	0.7	0.6	0.8	15.0	0.6	0.4	0.9	10.8	0.4	0.3	0.6
Haematological disorders	1.8	1.0	0.6	0.4	0.9	0.8	0.5	0.2	1.2	0.5	0.3	0.1	1.3	1.7	0.9	0.6	1.6	0.9	0.5	0.1	2.0	0.3	0.1	0.0	1.0
Injury or poisoning	18.2	14.8	0.8	0.7	0.9	13.3	0.7	0.6	0.9	13.2	0.7	0.5	1.0	17.7	1.0	0.8	1.1	11.2	0.6	0.4	0.9	13.1	0.7	0.5	0.9
Endocrine, nutrition, and metabolism diseases	8.4	5.0	0.6	0.5	0.7	4.2	0.5	0.3	0.8	4.9	0.6	0.4	0.9	6.7	0.8	0.6	1.0	4.3	0.5	0.3	1.0	2.1	0.2	0.1	0.5
Diseases of the digestive system	12.8	6.9	0.5	0.5	0.6	6.2	0.5	0.3	0.7	8.5	0.7	0.5	1.0	7.7	0.6	0.5	0.8	6.4	0.5	0.3	0.8	4.6	0.4	0.2	0.6
Diseases of the genitourinary system	10.1	7.8	0.8	0.7	0.9	7.8	0.8	0.6	1.1	8.5	0.8	0.6	1.2	8.7	0.9	0.7	1.1	5.6	0.5	0.3	1.0	5.9	0.6	0.4	0.9
Infectious diseases	7.6	4.9	0.7	0.5	0.8	4.4	0.6	0.4	0.9	4.9	0.7	0.4	1.0	6.5	0.9	0.7	1.1	4.7	0.6	0.3	1.1	1.8	0.2	0.1	0.5
Bone, muscle, and connective tissues diseases	1.3	0.6	0.4	0.3	0.8	0.2	0.2	0.0	1.1	1.1	0.9	0.3	2.4	0.5	0.4	0.2	1.0	0.4	0.3	0.0	2.5	0.8	0.6	0.2	1.9
Neoplasms	6.5	4.3	0.7	0.5	0.8	1.0	0.2	0.1	0.4	4.1	0.6	0.4	1.1	6.2	0.9	0.7	1.2	4.3	0.7	0.4	1.2	3.9	0.6	0.4	1.0
Diseases of the nervous system	3.5	2.3	0.7	0.5	0.9	2.2	0.7	0.4	1.2	0.5	0.2	0.0	0.7	3.2	1.0	0.7	1.4	2.1	0.6	0.3	1.6	1.8	0.5	0.3	1.1
Mental and behavioural disorders	2.5	1.7	0.7	0.5	0.9	1.8	0.7	0.4	1.4	1.4	0.5	0.2	1.3	1.5	0.6	0.3	1.0	2.1	0.9	0.4	2.1	2.1	0.8	0.4	1.7
Respiratory diseases	42.7	30.4	0.7	0.7	0.8	37.6	0.9	0.8	1.0	23.6	0.6	0.4	0.7	29.0	0.7	0.6	0.8	31.7	0.7	0.6	0.9	30.6	0.7	0.6	0.9
Other causes	5.8	4.1	0.7	0.6	0.9	4.8	0.8	0.6	1.3	4.9	0.9	0.5	1.4	3.8	0.7	0.5	0.9	3.0	0.5	0.2	1.1	3.6	0.6	0.4	1.1

### Hospital admission rates for major disease groups

**Fig. 2:**
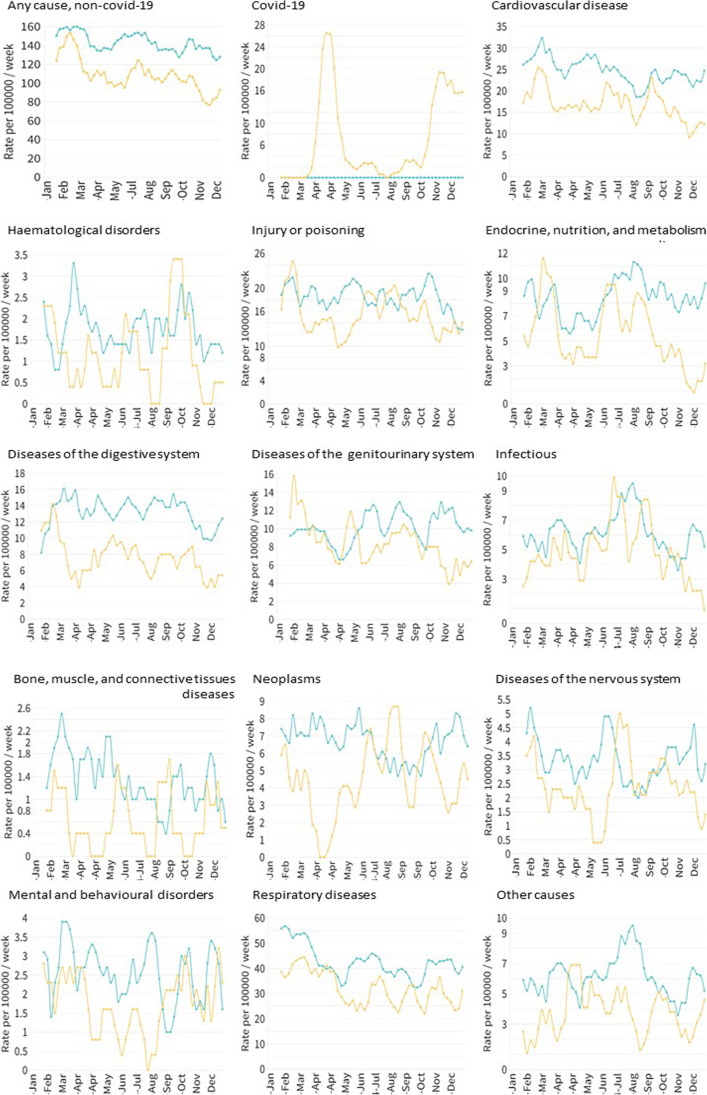
Weekly hospital admission rates by ICD-9-CM (international classification of diseases version 10) among LTCF residents in Tuscany and Apulia. *Blue line*: 2018–2019 average rate; *yellow line*: 2020 rate

For all other major diagnosis groups, the hospital admission rate decreased markedly during the pandemic period compared with the baseline period. The decreased rate remained statistically significant in all the pandemic phases only for hospital admission with a cardiovascular disease, endocrine, nutrition, and metabolism diseases and respiratory diseases diagnosis (Table 2, Fig. 2).

### Mortality among LTCF residents in Tuscany

The overall weekly average mortality rate for 1,000 LTCF residents in Tuscany was 8.9 before the start of the pandemic, and 12.5 during the pandemic. The proportion of deaths at 30 days after hospitalisation was 40.3% in the pre-pandemic period, and decreased to 34.5% in the pandemic period, excluding Covid-19 related hospitalisation. The overall 30-day mortality risk ratios among people admitted to hospital for non-Covid-19 causes increased during the first national lockdown (1.4, 1.2 to 1.7), during the period with new restrictions (1.5, 1.2 to 1.9), and during the regional lockdown (1.5, 1.2 to 1.9) compared with the pre-pandemic baseline period. The increase in mortality risk was higher among males than females for the first pandemic phases, while we have the opposite scenario in the last reported pandemic phases (Table 3).

**Table 3:** Thirty-day mortality risk and risk ratio among LTCF residents admitted to hospital for non-Covid-19 medical conditions during Covid-19 pandemic in Tuscany compared with the pre-pandemic baseline period, 2018–2020

	Baseline (reference)	Covid-19 pandemic period				First national lockdown				Gradual reopening phase				Few restrictions				New restrictions				Lockdowns on a regional basis			
	Risk	Risk	HR	(95% CI)		Risk	HR	(95% CI)		Risk	HR	(95% CI)		Risk	HR	(95% CI)		Risk	HR	(95% CI)		Risk	HR	(95% CI)	
Any cause (including COVID-19)	21.9	29.1	1.4	1.3	1.5	32.2	1.6	1.3	1.9	27.5	1.2	1.0	1.6	23.5	1.1	0.9	1.2	40.1	2.0	1.6	2.4	31.5	1.8	1.5	2.2
Any cause (excluding COVID-19)	21.9	26.4	1.2	1.1	1.4	29.7	1.4	1.2	1.7	26.7	1.2	0.9	1.5	23.6	1.1	0.9	1.2	33.0	1.5	1.2	1.9	26.1	1.5	1.2	1.9
Male	22.6	27.9	1.3	1.1	1.5	33.1	1.6	1.2	2.1	32.2	1.5	1.0	2.2	24.3	1.1	0.9	1.4	29.3	1.3	0.9	2.0	26.8	1.4	1.0	2.1
Female	21.5	25.4	1.2	1.1	1.4	27.2	1.3	1.0	1.7	23.6	1.0	0.8	1.5	23.2	1.0	0.9	1.3	35.5	1.7	1.2	2.3	25.7	1.6	1.1	2.1
ICD-9-CM																									
Cardiovascular disease	21.7	27.4	1.3	1.0	1.7	26.4	1.2	0.7	2.1	33.3	1.6	0.9	2.7	26.0	1.2	0.8	1.7	19.2	0.8	0.3	2.0	33.3	2.1	1.1	3.8
Haematological disorders	16.1	26.1	1.5	0.5	4.1	0.0	0.0	0.0	.	0.0	0.0	0.0	.	31.3	1.8	0.6	5.3	50.0	6.1	0.7	51.2	.			
Injury or poisoning	20.8	27.9	1.4	1.1	1.9	23.5	1.1	0.6	2.0	40.0	2.3	1.3	3.9	24.6	1.3	0.9	1.8	26.9	1.2	0.6	2.6	33.3	2.0	1.2	3.4
Endocrine, nutrition, and metabolism diseases	23.7	19.7	0.8	0.4	1.4	44.4	2.1	0.8	5.7	9.1	0.3	0.0	2.3	12.2	0.5	0.2	1.1	25.0	0.9	0.1	6.2	50.0	4.2	1.3	13.3
Diseases of the digestive system	10.2	12.3	1.0	0.6	1.8	23.8	1.6	0.5	5.2	9.1	0.4	0.1	3.2	10.9	1.0	0.5	2.3	6.7	0.6	0.1	4.5	12.5	1.6	0.4	6.5
Diseases of the genitourinary system	19.6	15.6	0.8	0.5	1.3	34.5	2.1	1.1	4.0	12.0	0.6	0.2	1.9	10.3	0.5	0.2	1.0	18.2	0.9	0.2	3.8	12.5	0.8	0.3	2.6
Infectious diseases	30.0	28.4	1.0	0.6	1.5	41.2	1.6	0.7	3.3	26.7	0.9	0.3	2.4	20.4	0.7	0.3	1.2	28.6	0.9	0.2	3.8	57.1	2.9	1.1	7.9
Bone, muscle, and connective tissues diseases	9.4	0.0	0.0	0.0	1.0	.				0.0	0.0	1.0	0.0	0.0	0.0	1.0	0.0	0.0	0.0	1.0	0.0	0.0	0.0	1.0	0.0
Neoplasms	16.7	17.6	1.1	0.6	1.9	0.0	0.0	0.0	.	9.1	0.5	0.1	3.9	25.5	1.5	0.8	2.9	10.0	0.6	0.1	4.1	6.7	0.5	0.1	4.0
Diseases of the nervous system	12.1	22.7	2.2	1.0	4.7	16.7	1.6	0.2	12.2	0.0	0.0	0.0	.	22.2	2.2	0.8	5.5	33.3	2.6	0.3	19.7	33.3	3.8	0.9	16.5
Mental and behavioural disorders	13.6	3.6	0.3	0.0	1.9	0.0	0.0	.	.	0.0	0.0	.	.	9.1	0.6	0.1	4.7	0.0	0.0	.	.	0.0	0.0	.	.
Respiratory diseases	27.4	34.1	1.3	1.1	1.5	36.1	1.3	1.0	1.8	32.4	1.2	0.8	1.8	32.9	1.2	1.0	1.5	47.9	2.0	1.4	2.8	25.9	1.2	0.8	1.8
Other causes	40.5	54.8	1.6	1.1	2.3	61.5	1.7	0.8	3.6	60.0	2.0	0.9	4.5	41.7	1.0	0.5	2.0	100.0	2.9	1.2	7.0	50.0	1.9	0.8	4.6

## Discussion

Although there is extensive literature describing Covid-19 outbreaks in LTCFs and the excess mortality that occurred during the pandemic (ECDC Public Health Emergency Team 2020; de Girolamo et al. 2020; Thompson et al. 2020), to our knowledge this is the first attempt to extensively study the indirect impact of the Covid-19 pandemic on hospital care provided to LTCFs residents in Europe.

Here, we analysed regional health registers that include residents of LTCFs in Tuscany and Apulia and observed a lower overall hospitalisation rate during the1st year of the pandemic compared with the pre-pandemic period, combined with an increased risk of mortality within 30 days of hospital admission.

During the study period, we observe that the weekly average population of the LTCFs gradually decreased, and this is in line with the Ministry of Health's recommendations (Istituto Superiore di Sanità 2020b) to limit new admissions of residents to urgent and unavoidable cases, in order to allow for a reduction in the number of residents to facilitate management of Covid-19 cases in isolation. In these recommendations, it is suggested to avoid sending residents to hospitals for specialist visits and instrumental examinations as much as possible.

This may be one of the reasons why, in our study, we observe a significant reduction in hospital admission rate during the pandemic period compared to the previous 2 years. This reduction has also been reported in the general population in Italy as in other countries (Kalanj et al. 2021; Boldrini et al. 2021, Rennert-May et al. 2021; Shah et al. 2022; Spadea et al. 2021). Furthermore, our results are in line with the findings of Betini et al., who reported a drop by 27% of LTCF resident transfers to hospitals for the treatment of chronic conditions and infections (Betini et al. 2021).

According to our results, the decrease in the admission rate is particularly pronounced in the second epidemic wave. This may be due to the fact that, although the hospitals were better prepared, the second epidemic wave was more extensive than the first (Italian National Institute of Health (Istituto Superiore di Sanità) 2020). Moreover, teams of healthcare workers periodically accessed the LTCFs to deal mainly with Covid-19 patients, allowing the nursing home staff and general practitioners who periodically visit the facilities to devote themselves to other patients (Istituto Superiore di Sanità 2020b).

The magnitude of the reduction in the hospitalisation rate for the major disease groups was homogenous (around 30–40%) if we compare the pre-pandemic period with the pandemic period as a whole. In specific periods, the reduction in the rate of hospitalisation for specific diseases was more pronounced. For instance, for neoplasms during the first lock-down the reduction in hospitalisation rates is about 80%, about twice the reduction reported for the same period in the Italian population (Spadea et al. 2021). The two Italian regions in our study have different hospitalisation rates for both the baseline and pandemic periods. In particular, the Apulia region reported lower hospitalisation rates and has experienced a more drastic reduction in access to hospital during the pandemic. This may be due to various factors: the healthcare provided in LTCFs may be dissimilar in the two regions, or criteria for hospitalisation may be different. Further studies are needed for a proper understanding of the reasons behind these differences as well as a harmonisation of data collection systems.

During the pandemic period, the mortality risk at 30 days after non-Covid-19 hospital admission increased by about 20%, higher when compared to age-specific mortality rates of the general population (Dorrucci et al. 2021), and this is consistent with what previous studies have reported (Berloto et al. 2020; Betini et al. 2021; Kalanj et al. 2021; Sepulveda et al. 2020).

Even before the crisis, the Italian LTCFs were characterized by weaknesses, due to a strong level of fragmentation both in terms of competencies among institutional and non-institutional actors, and unheard struggles to enter the policymakers’ agenda. LTCFs were not conceived and developed as a comprehensive model, rather they emerged from multiple legislative interventions that aimed intermittently at integrating what existed already (Berloto et al. 2020). LTCF governance structure is, at the central level, somewhere in the middle between the Ministry for Labour and Social Policy and the Ministry of Health.

Moreover, R&AP implement the dual ministerial policies by organising the LTCFs at the local level, defining the network of services; ultimately, local health authorities and municipalities manage services and interventions at the local and individual level. This fragmented situation is further compromised by the insufficient level of coordination that exists among all the actors involved in LTCFs: the absence of national awareness and lack of strategic vision inevitably inhibits dialogue, cooperation, and joint actions even in non-crisis times. This fragmentation also affects the information monitoring system, which is not integrated with national health data collection systems and is heterogeneous among regions, as evidenced by the fact that four out of the six regions that were invited to participate in the study declined because of low quality of data. However, although imperfect, these registers are available at regional level. Using them allows us not only to evaluate the health care performance of LTCFs, but also to advocate for the development of unified and comparable data collection systems across regions.

### Limits

This study has limitations. First, while we acknowledge that data regarding age-specific mortality, comorbidities, and cause of death of older residents would allow a more detailed assessment of the indirect impact of COVID-19 pandemic in LTCFs, we decided not to further stratify by age groups in order to increase accuracy of estimates. Second, the epidemic burden and organisation of LTCFs differ among Italian regions; therefore, it is not possible to translate our findings into the Italian context. Finally, we have no information on the type of LTCFs in which the subjects reside. This does not allow us to assess whether there are differences between privately and publicly managed LTCFs that might have had implications on the outcome during the Covid-19 pandemic.

## Conclusion

The Covid-19 pandemic had strong direct and indirect impact on LTCFs. They are vulnerable places for structural reasons and because of the residents’ characteristics; therefore, LTCFs need to be prioritized in pandemic preparedness plans.

In addition to the pandemic, it is important to monitor LTCFs potential threats and health performance. This implies integrating LTCF health registries within national surveillance and implementing possible sentinel sites for syndromic surveillance for early warning. These registries are available, and can be harmonised among regions and used to build a monitoring system against key indicators reflecting healthcare quality, but also to ensure appropriate use of the health system resources, including access to specialised services and hospitalisation.

## Supplementary information

ESM 1 (DOCX 201 kb)

## Data Availability

Data can be obtained upon request. Requests should be directed towards the corresponding author by email (giudittascardina77@gmail.com). Due to restrictions based on privacy regulations, data can not be made freely available in a public repository.
