# Supplementary material for: Hospital admission and mortality rates for non-Covid diseases among residents of the long-term care facilities before and during the pandemic: a cohort study in two Italian regions
Source: Z Gesundh Wiss. 2023 May 16:1–13. Online ahead of print. doi: 10.1007/s10389-023-01925-1 (PMC10185456; doi:10.1007/s10389-023-01925-1)

Supplemental material

***Table 1e.*** *Hospital admission rates (per 100 000/week) among LTCFs residents in Tuscany during covid-19 pandemic compared with pre-pandemic baseline period, 2018-2020.*

|  | Baseline (reference) | Covid-19 pandemic period | | | | First national lockdown | | | | Gradual reopening phase | | | | Few restrictions | | | | New restrictions | | | | Lockdowns on regional basis | | | |
| --- | --- | --- | --- | --- | --- | --- | --- | --- | --- | --- | --- | --- | --- | --- | --- | --- | --- | --- | --- | --- | --- | --- | --- | --- | --- |
|  | Rate | Rate | RR | (95% CI) | | Rate | RR | (95% CI) | | Rate | RR | (95% CI) | | Rate | RR | (95% CI) | | Rate | RR | (95% CI) | | Rate | RR | (95% CI) | |
| Any cause  (including COVID-19) | 155.8 | 146.9 | 0.9 | 0.9 | 1.0 | 155.8 | 1.0 | 0.9 | 1.1 | 111.9 | 0.7 | 0.6 | 0.8 | 141.0 | 0.9 | 0.8 | 1.0 | 169.4 | 1.1 | 1.0 | 1.2 | 168.3 | 1.1 | 1.0 | 1.2 |
| Any cause  (excluding COVID-19) | 155.8 | 123.5 | 0.8 | 0.8 | 0.8 | 114.9 | 0.7 | 0.7 | 0.8 | 107.2 | 0.7 | 0.6 | 0.8 | 137.9 | 0.9 | 0.8 | 0.9 | 117.6 | 0.8 | 0.6 | 0.9 | 115.9 | 0.7 | 0.7 | 0.8 |
| Male | 214.7 | 178.2 | 0.8 | 0.8 | 0.9 | 182.9 | 0.9 | 0.7 | 1.0 | 147.0 | 0.7 | 0.6 | 0.8 | 194.1 | 0.9 | 0.8 | 1.0 | 178.4 | 0.8 | 0.7 | 1.1 | 160.5 | 0.7 | 0.6 | 0.9 |
| Female | 133.8 | 103.6 | 0.8 | 0.7 | 0.8 | 89.7 | 0.7 | 0.6 | 0.8 | 92.9 | 0.7 | 0.6 | 0.8 | 117.4 | 0.9 | 0.8 | 1.0 | 95.3 | 0.7 | 0.6 | 0.9 | 99.7 | 0.7 | 0.6 | 0.9 |
| ICD-10 |  |  |  |  |  |  |  |  |  |  |  |  |  |  |  |  |  |  |  |  |  |  |  |  |  |
| Cardiovascular disease | 25.2 | 18.1 | 0.7 | 0.6 | 0.8 | 18.6 | 0.7 | 0.6 | 0.9 | 18.2 | 0.7 | 0.5 | 1.0 | 20.3 | 0.8 | 0.7 | 1.0 | 16.7 | 0.7 | 0.4 | 1.0 | 13.0 | 0.5 | 0.4 | 0.7 |
| Haematological disorders | 1.9 | 1.4 | 0.8 | 0.5 | 1.2 | 0.9 | 0.5 | 0.2 | 1.5 | 0.8 | 0.5 | 0.1 | 1.9 | 2.5 | 1.3 | 0.8 | 2.3 | 1.4 | 0.8 | 0.2 | 3.1 | 0.0 | 0.0 | 0.0 | . |
| Injury or poisoning | 17.6 | 19.2 | 1.1 | 1.0 | 1.2 | 16.4 | 0.9 | 0.7 | 1.2 | 15.7 | 0.9 | 0.6 | 1.2 | 22.2 | 1.3 | 1.1 | 1.5 | 14.7 | 0.8 | 0.5 | 1.3 | 20.7 | 1.2 | 0.9 | 1.6 |
| Endocrine, nutrition, and metabolism diseases | 8.3 | 4.6 | 0.6 | 0.4 | 0.7 | 3.3 | 0.4 | 0.2 | 0.7 | 4.7 | 0.6 | 0.3 | 1.0 | 6.3 | 0.8 | 0.6 | 1.1 | 2.8 | 0.3 | 0.1 | 0.9 | 2.6 | 0.3 | 0.1 | 0.7 |
| Diseases of the digestive system | 14.3 | 9.0 | 0.6 | 0.5 | 0.8 | 7.9 | 0.6 | 0.4 | 0.8 | 9.3 | 0.7 | 0.4 | 1.0 | 10.7 | 0.7 | 0.6 | 1.0 | 7.6 | 0.5 | 0.3 | 1.0 | 6.5 | 0.5 | 0.3 | 0.8 |
| Diseases of the genitourinary system | 11.4 | 10.9 | 1.0 | 0.8 | 1.1 | 10.6 | 0.9 | 0.7 | 1.3 | 11.0 | 1.0 | 0.7 | 1.4 | 12.2 | 1.1 | 0.8 | 1.4 | 8.4 | 0.7 | 0.4 | 1.3 | 9.1 | 0.8 | 0.5 | 1.2 |
| Infectious diseases | 8.8 | 5.9 | 0.7 | 0.5 | 0.8 | 5.1 | 0.6 | 0.4 | 1.0 | 6.4 | 0.7 | 0.4 | 1.2 | 7.7 | 0.9 | 0.7 | 1.2 | 4.2 | 0.5 | 0.2 | 1.1 | 3.0 | 0.3 | 0.2 | 0.7 |
| Bone, muscle, and connective tissues diseases | 1.1 | 0.4 | 0.4 | 0.2 | 0.8 | 0.0 | 0.0 | 0.0 | . | 0.4 | 0.4 | 0.1 | 2.7 | 0.6 | 0.5 | 0.2 | 1.5 | 0.7 | 0.6 | 0.1 | 4.4 | 0.4 | 0.4 | 0.1 | 2.7 |
| Neoplasms | 7.1 | 5.5 | 0.8 | 0.6 | 1.0 | 0.9 | 0.1 | 0.0 | 0.4 | 4.7 | 0.7 | 0.4 | 1.2 | 7.6 | 1.1 | 0.8 | 1.4 | 5.5 | 0.8 | 0.4 | 1.6 | 6.5 | 0.9 | 0.5 | 1.5 |
| Diseases of the nervous system | 3.2 | 2.8 | 0.9 | 0.6 | 1.2 | 2.1 | 0.7 | 0.3 | 1.4 | 0.8 | 0.3 | 0.1 | 1.1 | 4.2 | 1.3 | 0.9 | 2.0 | 2.1 | 0.7 | 0.2 | 2.1 | 2.5 | 0.8 | 0.4 | 1.8 |
| Mental and behavioural disorders | 2.5 | 1.8 | 0.7 | 0.5 | 1.0 | 1.8 | 0.7 | 0.3 | 1.6 | 1.3 | 0.5 | 0.2 | 1.6 | 1.7 | 0.7 | 0.4 | 1.2 | 1.4 | 0.6 | 0.1 | 2.2 | 2.6 | 1.0 | 0.5 | 2.3 |
| Respiratory diseases | 49.0 | 40.2 | 0.8 | 0.7 | 0.9 | 42.9 | 0.9 | 0.1 | 1.0 | 29.2 | 0.6 | 0.5 | 0.8 | 39.0 | 0.8 | 0.7 | 0.9 | 48.7 | 1.0 | 0.8 | 1.3 | 44.5 | 0.9 | 0.7 | 1.1 |
| Other causes | 5.7 | 4.0 | 0.7 | 0.5 | 0.9 | 4.3 | 0.8 | 0.4 | 1.3 | 4.7 | 0.8 | 0.5 | 1.5 | 3.6 | 0.6 | 0.4 | 1.0 | 3.5 | 0.6 | 0.3 | 1.5 | 4.4 | 0.8 | 0.4 | 1.4 |

***Table e2.*** *Hospital admission rates (per 100 000/week) among LTCFs residents in Apulia during covid-19 pandemic compared with pre-pandemic baseline period, 2018-2020.*

|  | Baseline (reference) | Covid-19 pandemic period | | | | First national lockdown | | | | Gradual reopening phase | | | | Few restrictions | | | | New restrictions | | | | Lockdowns on regional basis | | | |
| --- | --- | --- | --- | --- | --- | --- | --- | --- | --- | --- | --- | --- | --- | --- | --- | --- | --- | --- | --- | --- | --- | --- | --- | --- | --- |
|  | Rate | Rate | RR | (95% CI) | | Rate | RR | (95% CI) | | Rate | RR | (95% CI) | | Rate | RR | (95% CI) | | Rate | RR | (95% CI) | | Rate | RR | (95% CI) | |
| Any cause  (including COVID-19) | 114.5 | 65.0 | 0.6 | 0.5 | 0.6 | 91.1 | 0.8 | 0.7 | 0.9 | 68.2 | 0.6 | 0.5 | 0.7 | 63.7 | 0.6 | 0.5 | 0.6 | 52.5 | 0.5 | 0.3 | 0.6 | 42.5 | 0.4 | 0.3 | 0.5 |
| Any cause  (excluding COVID-19) | 114.5 | 56.8 | 0.5 | 0.5 | 0.5 | 70.2 | 0.6 | 0.5 | 0.7 | 64.4 | 0.6 | 0.5 | 0.7 | 59.5 | 0.5 | 0.5 | 0.6 | 45.8 | 0.4 | 0.3 | 0.6 | 31.1 | 0.3 | 0.2 | 0.4 |
| Male | 160.2 | 78.3 | 0.5 | 0.4 | 0.6 | 90.5 | 0.6 | 0.4 | 0.8 | 79.3 | 0.5 | 0.3 | 0.7 | 89.5 | 0.6 | 0.5 | 0.7 | 67.6 | 0.4 | 0.3 | 0.7 | 42.6 | 0.3 | 0.2 | 0.4 |
| Female | 95.6 | 46.8 | 0.5 | 0.4 | 0.6 | 63.4 | 0.7 | 0.5 | 0.8 | 59.2 | 0.6 | 0.5 | 0.8 | 46.2 | 0.5 | 0.4 | 0.6 | 35.0 | 0.4 | 0.2 | 0.6 | 25.3 | 0.3 | 0.2 | 0.4 |
| ICD-10 |  |  |  |  |  |  |  |  |  |  |  |  |  |  |  |  |  |  |  |  |  |  |  |  |  |
| Cardiovascular disease | 22.8 | 11.9 | 0.7 | 0.5 | 0.8 | 12.2 | 0.5 | 0.4 | 0.8 | 12.4 | 0.6 | 0.3 | 0.9 | 13.2 | 0.6 | 0.4 | 0.8 | 12.3 | 0.5 | 0.3 | 1.0 | 7.6 | 0.3 | 0.2 | 0.6 |
| Haematological disorders | 1.5 | 0.3 | 0.7 | 0.6 | 1.0 | 0.6 | 0.4 | 0.1 | 2.8 | 0.0 | 0.0 | 0.0 | . | 0.3 | 0.2 | 0.0 | 1.3 | 0.0 | 0.0 | 0.0 | . | 0.6 | 0.4 | 0.1 | 3.0 |
| Injury or poisoning | 20.2 | 7.5 | 0.6 | 0.5 | 0.7 | 7.5 | 0.4 | 0.2 | 0.7 | 8.5 | 0.4 | 0.2 | 0.8 | 9.8 | 0.5 | 0.3 | 0.7 | 5.6 | 0.3 | 0.1 | 0.7 | 1.9 | 0.1 | 0.0 | 0.3 |
| Endocrine, nutrition, and metabolism diseases | 4.6 | 3.7 | 0.5 | 0.6 | 1.2 | 5.8 | 0.7 | 0.4 | 1.3 | 5.4 | 0.6 | 0.3 | 1.3 | 7.3 | 0.8 | 0.6 | 1.3 | 6.7 | 0.8 | 0.3 | 1.7 | 1.3 | 0.1 | 0.0 | 0.6 |
| Diseases of the digestive system | 8.8 | 3.3 | 0.5 | 0.4 | 0.7 | 2.9 | 0.3 | 0.1 | 0.8 | 7.0 | 0.8 | 0.4 | 1.5 | 2.4 | 0.3 | 0.1 | 0.5 | 4.5 | 0.5 | 0.2 | 1.4 | 1.9 | 0.2 | 0.1 | 0.7 |
| Diseases of the genitourinary system | 6.9 | 2.4 | 0.8 | 0.3 | 0.7 | 2.3 | 0.3 | 0.1 | 0.9 | 3.9 | 0.6 | 0.2 | 1.4 | 2.7 | 0.4 | 0.2 | 0.7 | 1.1 | 0.2 | 0.0 | 1.2 | 1.3 | 0.2 | 0.0 | 0.7 |
| Infectious diseases | 4.5 | 3.2 | 0.5 | 0.6 | 1.2 | 2.9 | 0.7 | 0.3 | 1.6 | 2.3 | 0.5 | 0.2 | 1.7 | 4.4 | 1.0 | 0.6 | 1.7 | 5.6 | 1.3 | 0.5 | 3.2 | 0.0 | 0.0 | 0.0 | . |
| Bone, muscle, and connective tissues diseases | 1.6 | 0.8 | 0.5 | 0.3 | 0.7 | 0.6 | 0.4 | 0.1 | 2.8 | 2.3 | 1.5 | 0.5 | 5.0 | 0.3 | 0.2 | 0.0 | 1.3 | 0.0 | 0.0 | 0.0 | . | 1.3 | 0.8 | 0.2 | 3.5 |
| Neoplasms | 5.1 | 2.5 | 0.6 | 0.3 | 0.7 | 1.2 | 0.2 | 0.1 | 0.9 | 3.1 | 0.6 | 0.2 | 1.7 | 3.9 | 0.8 | 0.4 | 1.3 | 2.2 | 0.4 | 0.1 | 1.8 | 0.0 | 0.0 | 0.0 | . |
| Diseases of the nervous system | 3.8 | 1.4 | 0.9 | 0.4 | 0.9 | 2.3 | 0.6 | 0.2 | 1.7 | 0.0 | 0.0 | 0.0 | . | 1.6 | 0.4 | 0.2 | 1.0 | 2.2 | 0.6 | 0.1 | 2.4 | 0.6 | 0.2 | 0.0 | 1.2 |
| Mental and behavioural disorders | 2.5 | 1.5 | 0.7 | 0.7 | 1.2 | 1.7 | 0.7 | 0.2 | 2.3 | 1.5 | 0.6 | 0.2 | 2.6 | 1.1 | 0.4 | 0.2 | 1.2 | 1.3 | 1.4 | 0.4 | 4.4 | 1.3 | 0.5 | 0.1 | 2.1 |
| Respiratory diseases | 26.4 | 14.1 | 0.4 | 0.6 | 0.9 | 27.3 | 1.0 | 0.8 | 1.4 | 13.2 | 0.5 | 0.3 | 0.8 | 12.0 | 0.4 | 0.3 | 0.6 | 4.5 | 0.2 | 0.1 | 0.5 | 10.1 | 0.4 | 0.2 | 0.6 |
| Other causes | 6.1 | 4.3 | 0.8 | 0.6 | 1.1 | 5.8 | 1.0 | 0.5 | 1.8 | 5.4 | 0.9 | 0.4 | 1.9 | 4.4 | 0.7 | 0.4 | 1.2 | 2.2 | 0.4 | 0.1 | 1.5 | 2.5 | 0.4 | 0.2 | 1.1 |

***Figure 1e.*** *Overall non-Covid-19 (blue line) and Covid-19 (yellow line) hospital admission rates by gender and region.*


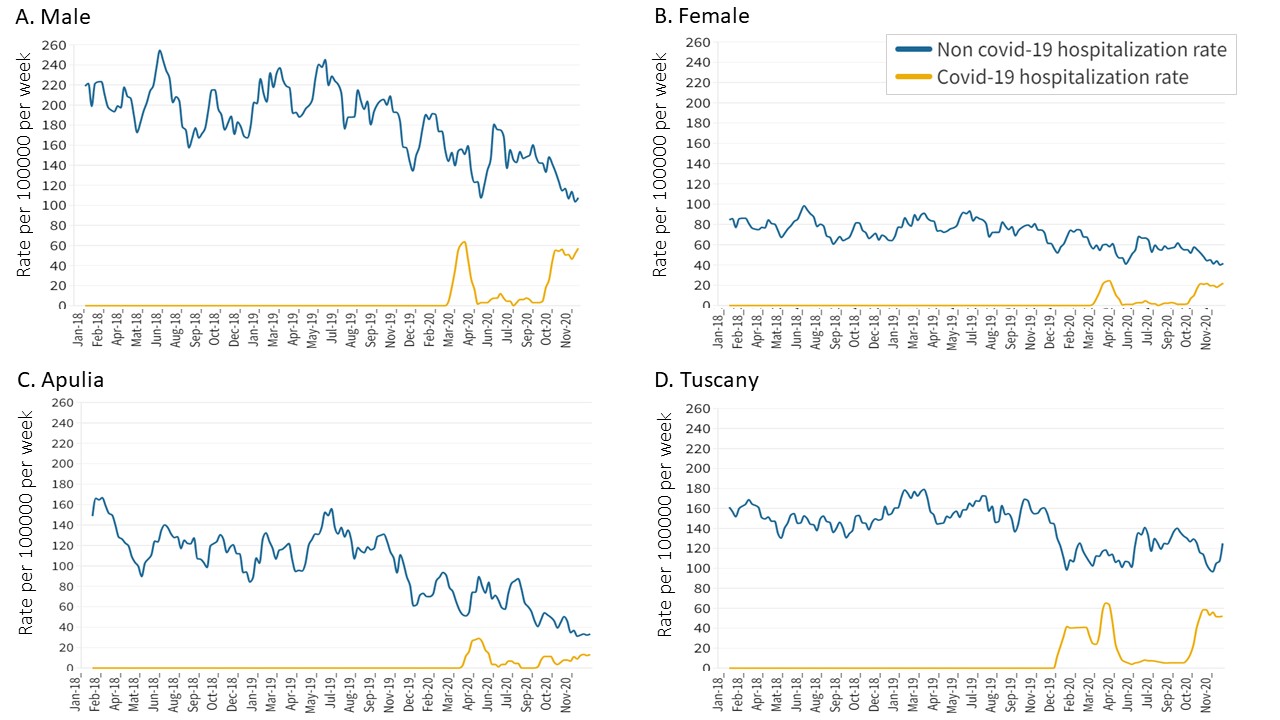

Supplement: Supplementary file 1 — (DOCX 201 kb) [file 10389_2023_1925_MOESM1_ESM.docx]
